# Modulation of GDF11 expression and synaptic plasticity by age and training

**DOI:** 10.18632/oncotarget.19854

**Published:** 2017-08-03

**Authors:** Emanuela De Domenico, Giovanna D’Arcangelo, Isabella Faraoni, Mattia Palmieri, Virginia Tancredi, Grazia Graziani, Paola Grimaldi, Lucio Tentori

**Affiliations:** ^1^ Department of Biomedicine and Prevention, University of Rome Tor Vergata, Rome, Italy; ^2^ Department of Systems Medicine, University of Rome Tor Vergata, Rome, Italy

**Keywords:** GDF11, skeletal muscle, hippocampus, exercise, long-term potentiation, Gerotarget

## Abstract

The Growth Differentiation Factor 11 (GDF11) has been controversially involved in the aging/rejuvenation process. To clarify whether GDF11 is differently expressed during aging, we have evaluated GDF11 levels in skeletal muscles and hippocampi of young and old mice, sedentary or subjected to a 12-weeks triweekly training protocol. The results of real-time PCR and Western blot analyses indicate that skeletal muscles of sedentary old mice express higher levels of GDF11 compared to young animals (*p* < 0.05). Conversely, in hippocampi no significant differences of GDF11 expression are detected. Analysis of long-term potentiation, a synaptic plasticity phenomenon, reveals that population spikes in response to a tetanic stimulus are significantly higher in sedentary young mice than in old animals (*p* < 0.01). Training induces a significant improvement of long-term potentiation in both young and old animals (*p* < 0.05), an increase (*p* < 0.05) of skeletal muscle GDF11 levels in young mice and a reduction of GDF11 expression in hippocampi of old mice (*p* < 0.05). Overall, data suggest that GDF11 can be considered an aging biomarker for skeletal muscles. Moreover, physical exercise has a positive impact on long-term potentiation in both young and old mice, while it has variable effects on GDF11 expression depending on age and on the tissue analyzed.

## INTRODUCTION

The growth differentiation factor 11 (GDF11) is a member of the transforming growth factor β (TGFβ) superfamily, homologous to another muscle-derived hormone, myostatin (MSTN). Although GDF11 and MSTN share 89% amino acid sequence identity within the mature C-terminal region [1], these proteins may have different functions. Similar to other TGFβ proteins, both GDF11 and MSTN are synthesized as precursor molecules and then processed into mature dimeric forms [2]. MSTN is expressed predominantly in skeletal muscle and plays an evolutionarily conserved role in antagonizing postnatal muscle growth. In fact, disruption of MSTN in many mammals (e.g. mice or cattle) causes muscle hypertrophy. In contrast, GDF11's functions in postnatal tissues are less known because of perinatal mortality of GDF11 KO animals. Nevertheless, various works have suggested a broader role of GDF11 in mammalian development and identified GDF11 as a hormonal regulator of different organs including brain and skeletal muscle [1, 3–9]. More recently it has been reported that overexpression of GDF11 in mice results in substantial atrophy of skeletal and cardiac muscle, inducing a cachexic phenotype not seen in mice expressing similar levels of MSTN [10].

Recently, studies have focused on the search for regulatory molecules that can reverse aging. Among these factors, GDF11 has been identified as a potential anti-aging candidate. However, some data on GDF11 expression and function are contradictory and GDF11 role in aging is still matter of lively debate. Indeed, initial studies in rodent models exploiting heterochronic parabiosis (in which circulatory systems of young and aged animals are connected) or using recombinant protein treatment, identified GDF11 as a molecule capable of rejuvenating cerebral, cardiac, skeletal muscle functions and attributed the diminished regenerative capacity of skeletal or cardiac muscle and brain of old mice to the decrease of GDF11 serum levels [11–13]. Afterwards, other reports questioned the age-related decline of circulating GDF11 and showed that GDF11 increases with age causing inhibition of muscle regeneration rather than fostering rejuvenation [14–18]. In addition, the specificity of antibodies and the methods used to detect the protein in previous studies have been criticized [14]. Therefore, further studies are needed to evaluate whether young and old individuals have a different GDF11 protein expression in tissues (e.g., skeletal muscle, hippocampus), and to clarify the actual role of GDF11 in the regulation of rejuvenation processes and longevity.

The increase of physical activity has been proposed as an effective therapeutic strategy to reduce the age-derived decline of muscular and cognitive functions although most of the molecular mechanisms underlying the benefit of exercise are still unknown. During the aging process exercise mediates beneficial effects on several brain functions by activating neurogenesis and delaying neurodegenerative processes [19, 20]. Recently, it has been reported that exercise mediates beneficial effects on brain plasticity and functions [6, 21–23]. Brain plasticity refers to the ability of the brain to modify its structure and function in response to maturation, learning, environmental stimuli or pathological state [24]. This activity-dependent phenomenon translates into a persistent boost in synaptic transmission, called long-term potentiation (LTP) that is considered the cellular and molecular substrate of learning and memory processes. Aging is a biological process associated with physiological cognitive decline; in particular, it can harm quality of life and result in deficits of declarative and working memory, spatial learning, and attention. Heterochronic parabiosis of young blood in old mice has been shown to enhance LTP and this effect has been attributed to the high GDF11 levels present in the blood of young mice [6, 25, 26].

The present study is an attempt to clarify, in a murine model, whether GDF11 expression in skeletal muscle and hippocampal tissues undergoes modulation during the aging process and whether training modulates GDF11 expression and LTP.

## RESULTS

### GDF11 expression in skeletal muscle and hippocampus of young and old mice

**Figure 1:**
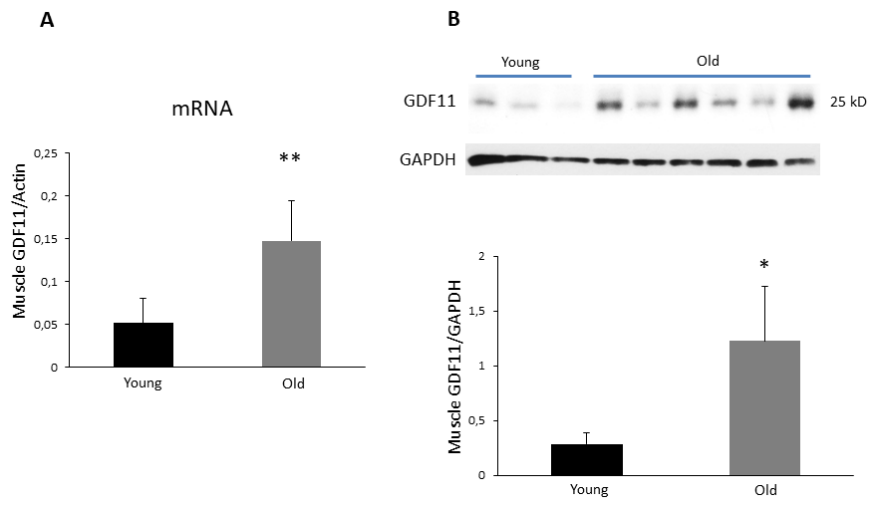
GDF11 mRNA and protein expression in skeletal muscles of young vs old male Balb/c mice Panel **A**. Analysis of GDF11 mRNA expression. Results of qRT-PCR are presented as relative expression of GDF11 mRNA values normalized to actin (*n* = 4 mice). **p < 0.01. Panel **B**. GDF11 protein expression in muscle tissue extracts obtained from 3 young and 6 old mice. Histogram represents the densitometric analysis of the immunoreactive bands and values are the mean optical densities (OD) +SD of GDF11 levels normalized to GAPDH. *p < 0.05.

Initially, we sought to investigate whether skeletal muscles of young or old Balb/c mice express different levels of GDF11. To this aim GDF11 mRNA levels from quadriceps muscle of young (3 months) and old (18 months) male animals were analyzed by quantitative real-time PCR (qRT-PCR). The results indicate that transcript levels in old mice were significantly higher in comparison to those detected in young mice (Figure 1A; *p* < 0.01). In order to validate the results of qRT-PCR, Western blot analysis of GDF11 expression was performed on protein extracts obtained from muscle tissues. The data show that old mice have a significant higher level of the 25 kD GDF11 mature form compared to their younger counterparts, in which the protein was barely detectable (Figure 1B; *p* < 0.05). Consistently, higher GDF11 expression levels were observed in old female Balb/c mice (Supplementary Figure 1S; *p* < 0.05). Similar differences in GDF11 mRNA or protein expression between young and old mice were detected also in male animals of a different strain (i.e., C57BL/6; Supplementary Figure 2S). Because of the concerns raised in previous studies regarding the lack of specificity of anti-GFD11 antibodies, the specificity of the anti-GDF11 monoclonal antibody used herein (i.e., clone 743833 R&D Systems) was verified by testing its ability to recognize two different commercially available recombinant GDF11 and MSNT proteins (i.e., Peprotech and R&D). The antibody specifically detected only GDF11 protein (Supplementary Figure 3S, panel A).

To confirm that the 25 kD band observed in immunoblot analysis using skeletal muscle lysates is not due to reaction of native murine immunoglobulins with the secondary anti-mouse antibody used for the detection of GDF11, the membrane was incubated with the secondary antibody only. In these experimental conditions no 25 kD bands were observed, confirming that the immunoreactive band recognized by the anti-GDF11 antibody is not from native mouse immunoglobulin light chain reacting with the secondary antibody (Supplementary Figure 1S, panel B).

### Long-term potentiation (LTP) in young and old mice

**Figure 2:**
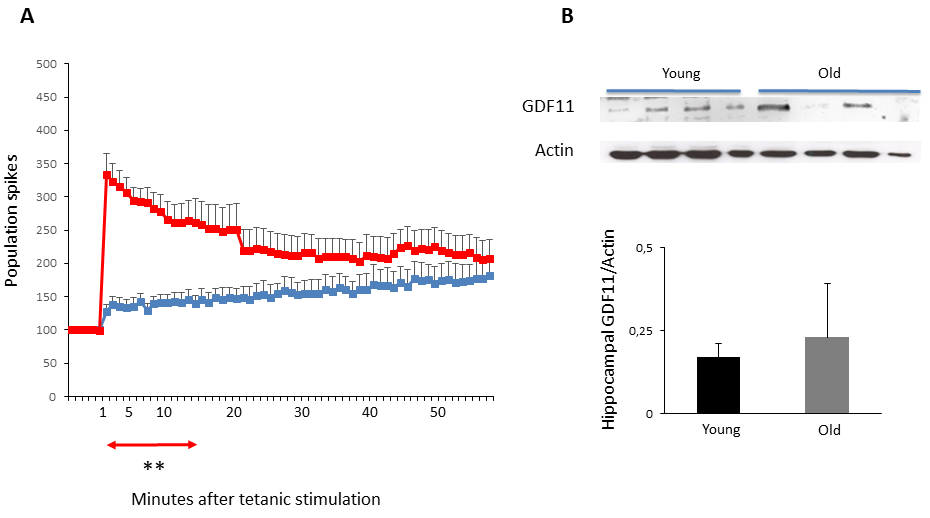
LTP and hippocampal GDF11 protein expression in young vs old mice Panel **A**. LTP at different times after tetanic stimulation in young (red) *vs* old (blue) mice. Values of the PS amplitude, calculated every minute, correspond to the average of six recordings/minute from 8 different slices of each group (4 mice/group). Differences between groups, from 1 up to 14 min after tetanic stimulation: **p < 0.01. Panel **B**. GDF11 expression in tissue extracts from hippocampi of young *vs* old sedentary mice (*n* = 4 for each group). Histogram represents the densitometric analysis of GDF11 protein levels and values are the mean OD +SD of GDF11 levels normalized to actin. Differences were not statistically significant.

Heterochronic parabiosis of young blood in old mice has been shown to enhance LTP that is a putative functional correlate of synaptic plasticity, learning and memory, and this effect has been attributed to the high GDF11 levels present in the blood of young mice [6, 25, 26]. Actually, among its pleiotropic properties, GDF11 increases neurogenesis, CNS plasticity and vasculature brain modeling [13]. In order to verify whether aging may affect LTP in mice, electrophysiological recordings on hippocampal slices from young and old Balb/c mice were analyzed. In samples from young mice a tetanic stimulus induced a sustained enhancement of population spikes (PS) that were significantly higher when compared to those of old animals (*p* < 0.05 from 1 to 14 min) (Figure 2A). Within the same time frame, also differences between LTP areas under the curve (AUC) of old and young animals were statistically significant (*p* < 0.001). On the contrary, a blockade of the induction of LTP was observed in old mice (Figure 2A), while no significant differences in the maintenance phase with respect of young animals were detected (values at different times after tetanic stimulation are reported in Table 1). In an attempt to correlate LTP responses to the levels of GDF11 protein expression, immunoblot analysis was performed in samples obtained from hippocampal tissues of young and old animals. The results revealed similar low levels of GDF11 in young animals, while a wide variation of GDF11 protein expression was observed in old mice, being barely visible in some samples and highly expressed in others (Figure 2B).

**Table 1:** Population spikes recorded after tetanic stimulation

Min	Young sedentary	Young trained	Old sedentary	Old trained
1	332.8± 32.0	343.8 ± 40.9	127.2±11.2	259.4± 23.0
5	294.3±19.9	390.2 ± 48.9	135.4±13.0	230.8 ± 23.2
10	266.5±27.1	383.8 ± 51.3	140.4±13.4	218.6 ± 23.6
20	251.1±38.5	326.6 ± 42.1	146.5±16.1	197.2 ± 19.1
30	217.0±28.0	345.2 ± 59.4	154.3±20.4	193.7 ± 19.4
40	209.8±30.8	322.7 ± 65.5	168.8±29.7	199.2 ± 23.9
50	219.5±29.2	307.5 ± 62.6	174.7±25.0	181.1 ± 21.5
60	211.8±30.1	290.9 ± 54.9	181.6±28.5	178.0 ± 23.4

**Figure 3:**
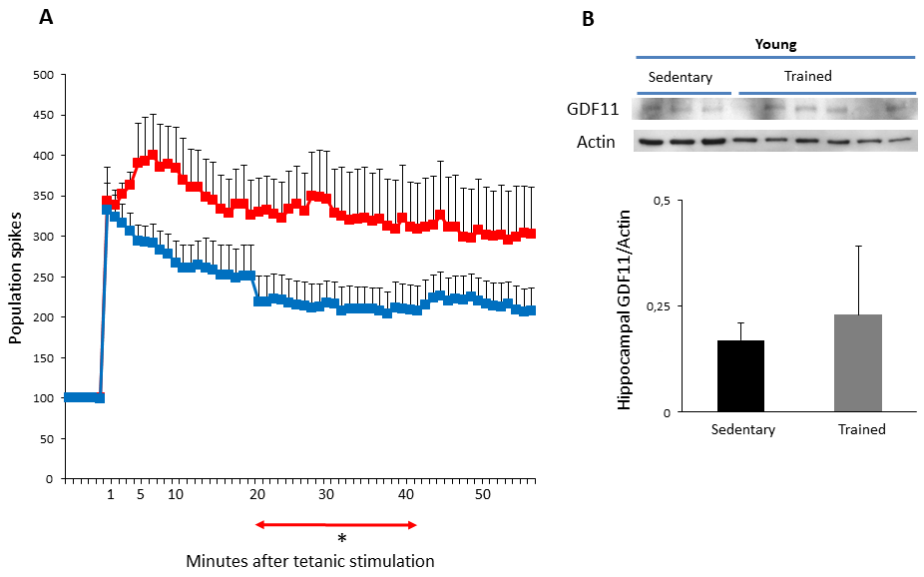
LTP and GDF11 protein expression in hippocampi of sedentary vs trained young mice Panel **A**. LTP at different times after tetanic stimulation in young sedentary (blue) *vs* young trained (red) mice. Values of the PS amplitude, calculated every minute, correspond to the average of six recordings/minute from 8 different slices of each group (6 mice/group). Differences between mean increment of PS values at different times after tetanic stimulation of sedentary *vs* trained mice: *p < 0.05 from 20 to 40 min. Panel **B**. GDF11 expression in tissue extracts from hippocampi of young sedentary (*n* = 3) *vs* trained (*n* = 6) mice. Histogram represents densitometric analysis of GDF11 protein levels and values are the mean OD +SD of GDF11 levels normalized to actin. Differences were not statistically significant.

Electrophysiological recording was performed on hippocampal slices from sedentary and trained young and old Balb/c mice. Values represent the mean of population spikes ±SD obtained after tetanic stimulation at the indicated times (see also Figures 3 and 5). Differences between groups are statistically significant for age (*p* <0.05) and training (*p* <0.05) as assessed by Two-Factor Repeated Measures ANOVA.

### Effect of training on body weight, glucose, cholesterol, triglycerides, lactate dehydrogenase in young and old mice

The effects of training on body weight and on blood parameters were evaluated in mice trained 3 times/week for 12 weeks as described in the Methods section. At the end of training period, animals were weighted and serum levels of glucose, cholesterol, triglycerides, lactate dehydrogenase (LDH) compared to those of sedentary controls. The results showed that young trained mice weighted significantly more than same-age sedentary controls at the end of training, whereas no differences in weight were observed after training when old mice were compared to their sedentary controls. In young trained mice glucose, cholesterol and LDH levels were significantly lower than those detected in their sedentary counterparts, whereas no substantial changes in the levels of triglycerides were found between trained and untrained mice. Differently, in old mice, only glucose and LDH levels were significantly reduced after training, while the other parameters did not substantially change (Table 2).

**Table 2:** Effect of training on body weight and blood chemistry

YOUNG	SEDENTARY		TRAINED		
	MEAN^a^	SD	MEAN^a^	SD	t test^b^ (P)
**WEIGHT** (g)	20.6	3.7	25.6	2.1	0.01
**GLUCOSE** (mg/dl)	124.8	25.9	96.0	10.1	0.04
**CHOLESTEROL** (mg/dl)	151.2	30.3	110.0	12.4	0.04
**TRIGLYCERIDES** (mg/dl)	179.0	2.0	171.0	26.6	NS
**LDH** (U/l)	900.0	316.6	431.0	79.0	0.02
**OLD**	**SEDENTARY**		**TRAINED**		
**WEIGHT** (g)	28.5	2.1	29.2	2.0	NS
**GLUCOSE** (mg/dl)	140.0	13.9	88.0	17.0	0.03
**CHOLESTEROL** (mg/dl)	149.0	14.0	112.0	28.3	NS
**TRIGLYCERIDES** (mg/dl)	121.3	50.6	152.0	5.7	NS
**LDH** (U/l)	613.0	99.0	517.3	34.0	0.02

^a^Values represent the mean (±SD) of body weight (*n* = 8), glucose, cholesterol, triglycerides and LDH (*n* = 4) in sedentary and trained Balb/c mice measured at the end of the 12 weeks training period.

The mean initial body weight before training was 13.4±1.8 and 25.6 ±1.9 of young and old mice, respectively.

^b^Student's *t*-test comparing sedentary *vs* trained mice at the end of the training period.

### Effects of training on LTP and on GDF11 protein expression in hippocampus and skeletal muscle of young mice

**Figure 4:**
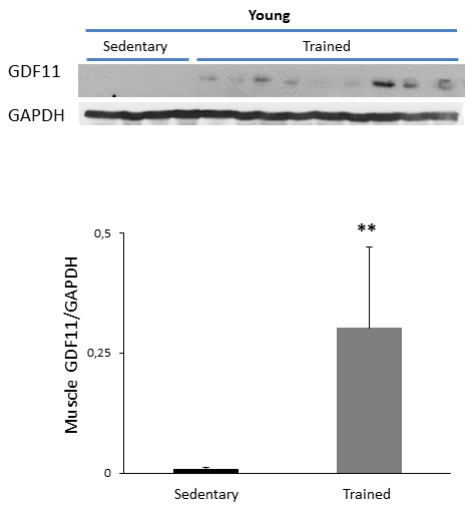
GDF11 expression in skeletal muscle extracts from sedentary vs trained young mice GDF11 protein expression in tissue muscle extracts from young sedentary (*n* = 4) and trained (*n* = 9) mice. Histogram represents the densitometric analysis of GDF11 protein and values are the mean OD +SD of GDF11 levels normalized to GAPDH bands. **p < 0.01.

Then, we tested if the adopted training protocol could influence synaptic plasticity, by means of LTP analysis. The results showed that training induced a significant increase in LTPs of young mice. Indeed, the PS values detected at different times, after tetanic stimulation, were significantly higher in trained mice in comparison to sedentary controls (*p* < 0.05 from 20 to 40 min) (Figure 3A and Table 1). In the attempt to correlate the effects of training on LTP response with GDF11 expression, at the end of training period, GDF11 protein expression was analyzed in extracts from hippocampal whole tissues of young mice and age-matched sedentary controls. The results indicate that GDF11 expression in hippocampi of trained young animals were variable (Figure 3B), whereas in skeletal muscle training induced a significant increase in the level of GDF11 (Figure 4, *p* < 0.001).

### Effects of training on LTP and GDF11 in hippocampus and skeletal muscle of old mice

**Figure 5:**
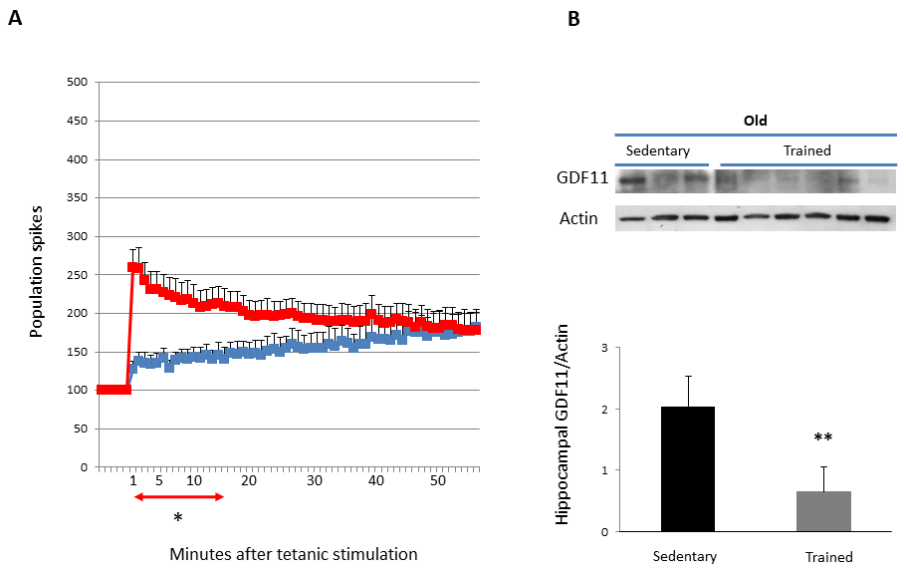
LTP and GDF11 protein expression in hippocampi of sedentary vs trained old mice Panel **A**. LTP at different times after tetanic stimulation in sedentary (blue) *vs* trained (red) old mice. Values of the PS amplitude, calculated every minute, correspond to the average of six recordings/minute (8 slices from 6 sedentary mice and 13 slices from 9 trained mice). Differences between mean increment of PS values at different times after tetanic stimulation of sedentary *vs* trained mice *p < 0.05 from 1-14 min. Panel **B**. GDF11 expression in tissue extracts from hippocampi of young sedentary (*n* = 3) *vs* trained mice (*n* = 6). Histogram represents the densitometric analysis of GDF11 protein levels and values are the mean OD +SD of GDF11 levels normalized to actin bands shown on panel **B**. **p < 0.01.

**Figure 6:**
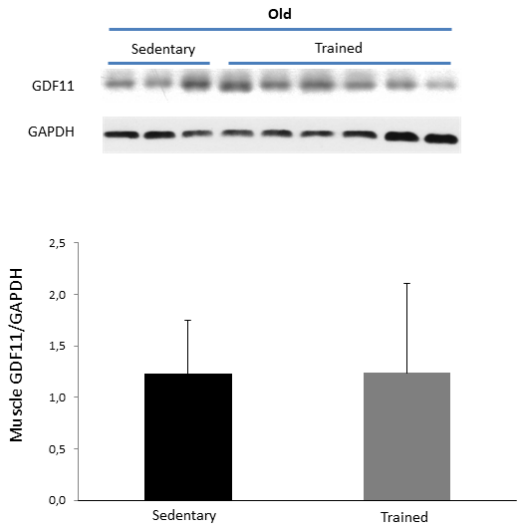
GDF11 protein expression in skeletal muscles of sedentary vs trained old mice GDF11 protein expression in tissue muscle extracts from young sedentary (*n* = 3) and trained mice (*n* = 6). Histogram represents the densitometric analysis of GDF11 protein and values are the mean OD +SD of GDF11 levels normalized to GAPDH bands. Differences were not statistically significant.

In old animals, analysis of synaptic plasticity after training showed a significant enhancement of LTP with respect to untrained controls of the same age, overcoming the block of the induction phase observed in same age sedentary mice (Figure 5A and Table 1). This increase was detected either examining the mean values of the PS obtained after tetanic stimulation or analyzing AUC at 1 up to 14 min time points (*p* < 0.05). Interestingly, analysis by Western blotting of GDF11 protein expression in extracts from hippocampi shows that GDF11 levels of trained animals were significantly lower than those of sedentary controls (*p* < 0.01) (Figure 5B). Conversely, in skeletal muscles no significant differences in GDF11 expression were found between sedentary and trained old mice (Figure 6).

**Figure 7:**
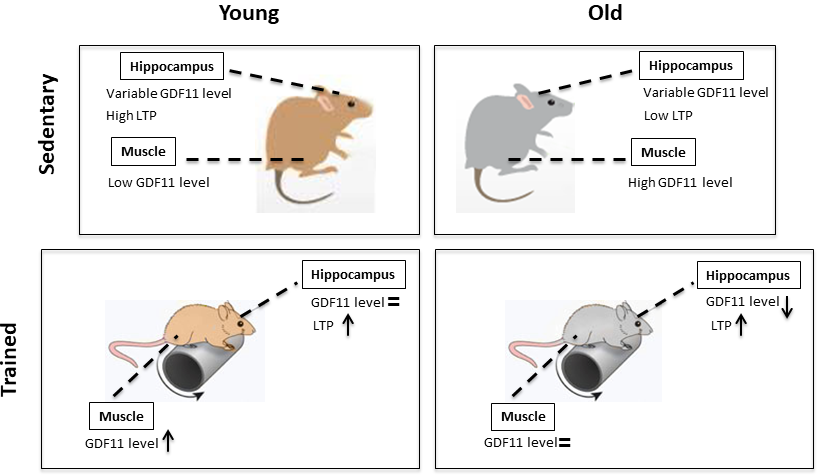
GDF11 expression levels and modulation of synaptic plasticity by age and training

## DISCUSSION

Nowadays, it is still controversial whether tissue levels of GDF11 protein expression are age-related. In the current study we provide evidence, by using an antibody which specifically recognizes GDF11 and does not cross react with MSTN, that this protein is expressed at higher levels in the skeletal muscle tissue of old mice compared to young animals independently of sex and strain. The results were also confirmed by quantitative analysis of GDF11 mRNA. This observation is in sharp contrast with studies showing GDF11 decline in skeletal muscle with age [12], but in agreement with others studies in which GDF11 protein expression was found to increase with age [14]. In addition, a recent report showed that supraphysiological levels of GDF11, obtained by virus-mediated protein overexpression, cause wasting of both skeletal and cardiac muscles [19]. The controversial results may reflect differences in experimental designs, strategies, detection reagents, specificity of GDF11 antibodies, sources of recombinant proteins used as controls [9, 15, 27, 28]. Our results, obtained with qRT-PCR using specific primers mapping in a GDF11 region which does not overlap with MSNT sequences and immunoblot analysis using an antibody specifically recognizing recombinant GDF11, indicate that skeletal muscles of old mice express higher GDF11 levels than young mice. The latter findings discourage the use of recombinant GDF11 to counteract age-related cardiac and skeletal muscle decline.

The age-dependent increase of GDF11 observed is limited to skeletal muscle; in fact, a wide variation of GDF11 protein expression was detected in the hippocampi of old animals. Actually, also in other tissues and in the serum the relationship between expression level and function of GDF11 is quite controversial. Recently GDF11 was reported to increase neurogenesis and to be involved in brain rejuvenation of aged mice [29]. Moreover, in a heterochronic parabiosis model, electrophysiological recordings on hippocampal slices of aged mice, showed that synaptic plasticity was enhanced by exposure to young blood [30]. Since GDF11 was found to be present at higher levels in the serum of young and heterochronic mice than in serum from old control animals, this protein was believed to be involved in the rejuvenating process. In addition, another study reported that recombinant GDF11 treatment improves the cerebral vasculature and enhances neurogenesis [13]. In the present study, we found variable levels of GDF11 in hippocampi of old mice with respect to those detected in young mice. Moreover, the GDF11 expression found in the hippocampi did not correlate with the impairment of synaptic plasticity in the hippocampal CA1 region, measured by LTP assay in old mice. At CA3-CA1 synapses, LTP induction is mainly due to N-methyl-D-aspartate receptors (NMDAR) activity. Electrophysiological studies consistently showed a decrease in the NMDAR component of the synaptic transmission in CA1 region during aging [31–34], partially related to oxidative stress, [33]. Indeed, in old animals we observed that a tetanic stimulus failed to induce a quick and sustained enhancement of PS that was instead detected in young mice.

Physical exercise has been proposed as an effective strategy to reduce the detrimental influence of aging on muscle and cognitive function. Indeed, it has been demonstrated that voluntary exercise can induce both structural and functional plasticity and can increase cell proliferation, neurogenesis, spatial learning and synaptic plasticity in both rats and mice [23, 35, 36]. In this study we sought to investigate whether the beneficial effects of a forced long-term specific training program (i.e., continuous progressive protocol, which can be appropriate also for aged animals) may result in modulation of GDF11 expression. The involvement of GDF11 in improving muscle features and increasing strength and endurance exercise capacity was suggested by studies showing that systemic supplementation of recombinant GDF11 reverses not only age-related cardiac hypertrophy, but also age-related skeletal muscle and stem cell dysfunction, and improves physical function in aged mice [12]. These effects were associated with an enhancement in the clearance of systemic lactate and lowering of glucose levels after exercise, suggesting an improved mitochondrial function [12]. In our model, training slightly but significantly increased GDF11 levels in skeletal muscles of young animals, but it did not affect protein expression in the same tissues of old mice. In hippocampal tissues training did not substantially affect GDF11 protein levels of young mice, whereas it significantly decreased GDF11 protein expression in old mice. Moreover, training significantly reduced serum glucose, LDH in both young and old animals and improved the weight in young animals, indicating beneficial effects of the continuous progressive training protocol. Actually, we recently demonstrated that a short-term aerobic exercise in adult mice enhances body weight and synaptic plasticity if training load is adequate and appropriate recovery periods are respected [37]. In the present work young animals, trained for a longer period (3 months), exhibit an enhancement in synaptic plasticity and old mice present a blockade reversal of the LTP induction phase indicating that motor activity exerts positive effects on neuronal plasticity. Since regular moderate aerobic exercise has been shown to promote antioxidant capacity on the brain [38], it can be hypothesized that this mechanism might be responsible to some extent of the LTP augmentation observed in trained old mice. Based on these results, the beneficial effects of training on synaptic plasticity did not consistently correlate with modifications of GDF11 expression in hippocampi.

In conclusion, this study suggests that differences in GDF11 expression vary depending on tissue localization. In fact, GDF11 expression in skeletal muscle increases with age, whereas its expression is variable in hippocampal tissues. Moreover, GDF11 appears to be differently modulated by training in skeletal muscle and hippocampi of young and old mice. Finally, positive LTP modulation can be considered a biological index of the beneficial effect of training on synaptic plasticity in aging (Figure 3).

## MATERIALS AND METHODS

### Animals, training, LTP and biochemistry analyses

Thirteen 3-months old (hereafter referred to as young) and fifteen 18-months old (hereafter referred to as old) Balb/c male mice were used for the analysis of GDF11 expression and LTP. Four young and four old female Balb/c mice or three young and three old male C57BL/6 mice (Charles River, Calco, Milan, Italy) were used for GDF11 expression analysis of skeletal muscle. For the training experiments young and old Balb/c male mice were each divided into two groups: one untrained group (hereafter referred to as sedentary control) and one group subjected to Rota-rod (Ugo Basile srl, Varese, Italy) training three times a week for a total of twelve weeks. Rota-Rod was utilized for the administration of the “continuous progressive protocol”, consisting in a progressive 2 RPM increase pace speed from 10 RPM to 32 RPM with 12 speed changes in 18 minutes.

At the end of the training period animal blood was collected from retro-orbital plexus and plasma separated for blood biochemistry. After at least a couple of days mice were anesthetized with halothane and sacrificed by decapitation. Then, quadriceps skeletal muscle and hippocampal tissues were collected and immediately frozen in liquid nitrogen to be successively processed. In addition, part of the tissues were placed in Trizol (Invitrogen, Groningen, The Netherlands) and frozen at -80 °C for molecular analysis of the transcripts by real time PCR. Some slices of hippocampus were further sectioned transversally with a chopper into thinner slices of 450 μm, transferred to a tissue chamber, placed in an interface between oxygenated Artificial Cerebrospinal Fluid (ACSF) and humidified gas (95% O_2_-5% CO_2_) at 32-34 °C (pH 7.4), constantly superfused at a flow rate of 1.2 ml/min with ACSF. For LTP, the extracellular recordings of PS and the subsequent analyses were made according to procedures previously indicated [39]. AUC within the indicated intervals was calculated using the function AUC of the package flux written with the programming language R.

All blood samples were microcentrifuged at 13,000 rpm for 7 min to separate the serum. Glucose, cholesterol, triglycerides, and LDH were measured using the automatic analyzer Keylab (BPC BioSed srl, Rome, Italy).

### Animal care and ethics statement

All procedures involving mice and care were conducted in accordance with the ethical standards, according to the Declaration of Helsinki, in compliance with our institutional animal care guidelines and following national and international directives (D.L. March 4, 2014, no. 26; directive 2010/63/EU of the European parliament and council; Guide for the Care and Use of Laboratory Animals, United States National Research Council, 2011). Experimental protocols were approved by the Animal Care and Use Committee at the institutions involved in this study and by the Italian Ministry of Health.

### Western blot analysis

Homogenates from hippocampus or skeletal muscle from hind limb were lysed in RIPA Buffer containing 1% NP-40, 50 mM Tris-HCl, pH 7.4, 150 mM NaCl + 2 mM EDTA in the presence of protease inhibitors (2 mM PMSF, 5 ug/mL leupeptin, 5 ug/mL pepstatin in ethanol). Equal amount of protein samples (80 μg) were separated by 12% sodium dodecyl sulfate-polyacrylamide gel under non reducing conditions and transferred to a PVDF membrane (Millipore, Billerica, MA). For immunodetection the following primary antibodies were used: monoclonal mouse IgG1 anti-GDF-11/BMP-11 mAb (Clone 743833 dilution 1:500; R&D Systems Inc., Minneapolis, MN), β-Actin or GAPDH as housekeeping controls (1:1000; Sigma-Aldrich, St. Louis, MO). The specific protein complexes formed upon addition of the anti-rabbit or anti-mouse secondary antibodies (1:5000; Cell Signaling Technology, Leiden, The Netherlands) were identified with the ECL reagents (GE healthcare, Milan, Italy). Optical densities (OD) were quantified using ImageJ freeware. Recombinant myostatin MSTN and GDF11 were purchased from Peprotech (Rocky Hill, NJ; cat. # 120-00 and 120-11, respectively) or R&D Systems (cat. # 788-G8/CF and 1958-GD). 100 ng of protein were resolved on 12% Bis-Tris mini gels.

### Real time PCR

Tissues were homogenized and total RNA extracted using TRIzol Reagent (Invitrogen) according to the manufacturer's instructions and 1 μg was retrotranscribed (RT), using M-MLV reverse transcriptase (Invitrogen). cDNA was used as template for qRT-PCR using SSOADV Universal SYBR Green (BioRad) in a PRISM 7300 Sequence Detection System (Applied Biosystems, Foster City, CA) and the following primers: forward, 5′-ACCCTGCAGTGCAGACAGAT -3′; reverse 5′-AACGTGAGTGTAGCTCAATCT -3′; for actin: forward: 5’-CTGTCGAGTCGCGTCCAC-3’; reverse: 5’-GCTTTGCACATGCCGGAG-3’ .

### Statistical analysis

Results were expressed as arithmetic mean ± standard deviation (SD). Statistical analysis of the differences between independent groups was performed by unpaired, two-tailed Student's *t*-test; *p* values < 0.05 (*), < 0.01 (**) and < 0.001 (***) were considered significant.

In Table 1 to determine whether statistical significant differences of population spikes occur between the groups examined, considering age and training as within and between factors, respectively, data were analyzed by Two-Factor Repeated Measures ANOVA using IBM SSPS statistics software.

## SUPPLEMENTARY FIGURES



## Abbreviations

GDF11: Growth Differentiation Factor 11
TGFβ: transforming growth factor β
MSTN: myostatin
qRT-PCR: quantitative real-time PCR
LTP: Long-term potentiation
PS: population spikes
LDH: lactate dehydrogenase
NMDAR: N-methyl-D-aspartate receptors
