# Supplementary material for: Modulation of GDF11 expression and synaptic plasticity by age and training
Source: Oncotarget. 2017 Aug 3;8(35):57991–8002. doi: 10.18632/oncotarget.19854 (PMC5601628; doi:10.18632/oncotarget.19854)
Supplement: Supplementary file 1 [file oncotarget-08-57991-s001.pdf]

Modulation of GDF11 expression and synaptic plasticity by age and training

SUPPLEMENTARY MATERIALS

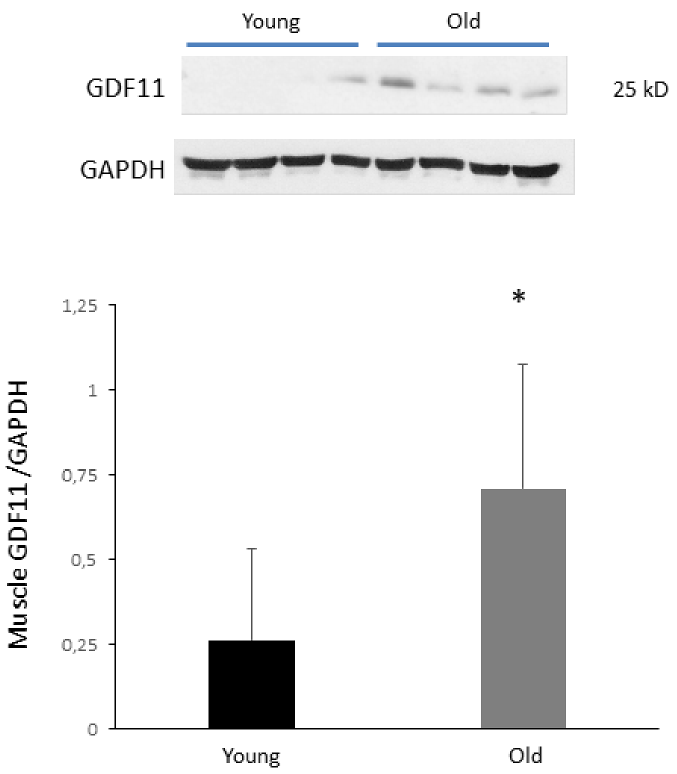

Supplementary Figure 1: GDF11 protein expression in muscles of young and old female Balb/c mice.
GDF11 protein expression in muscle tissue extracts obtained from young and old female Balb/c mice (n=4 for each group). Histogram represents the densitometric analysis of the immunoreactive bands and values are the mean OD +SD of GDF11 levels normalized to GAPDH. * $p<0.05$

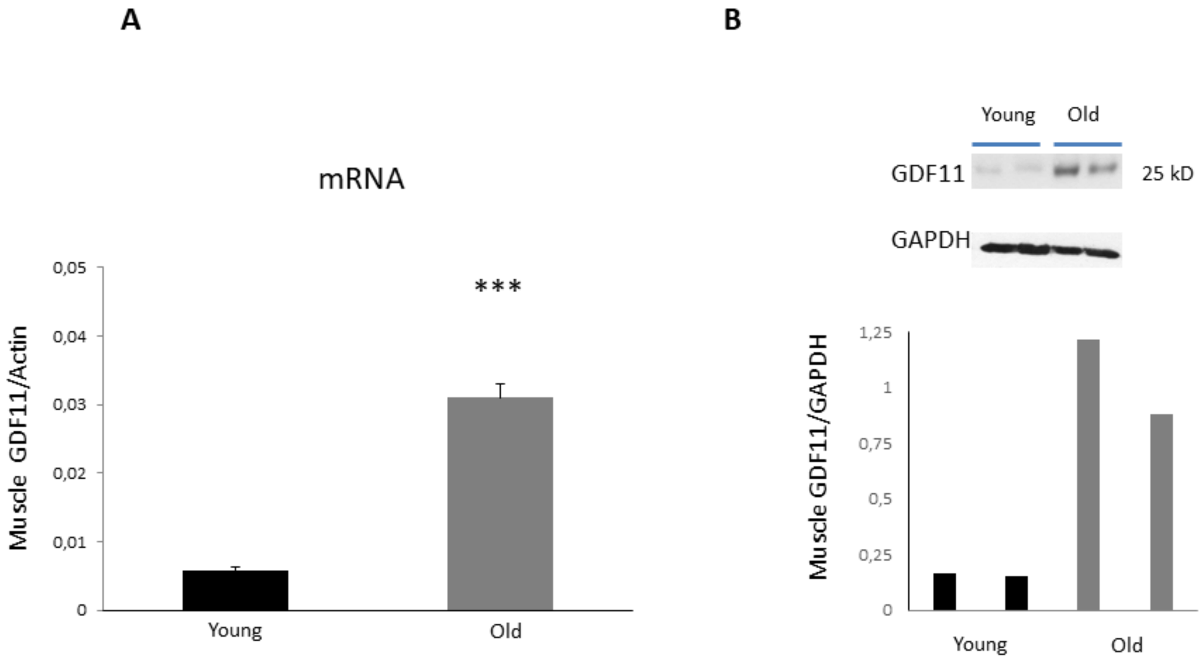

Supplementary Figure 2: GDF11 protein expression in skeletal muscles of male young and old male C57/B6J mice. Panel A GDF11 mRNA expression. Results of qRT-PCR are presented as the relative expression of GDF11 normalized to actin (n=3 mice). *** p<0.001

Panel B GDF11 protein expression in skeletal muscle tissue extracts obtained from young and old mice. Histogram represents the densitometric analysis of the GDF11 immunoreactive bands normalized to GAPDH.

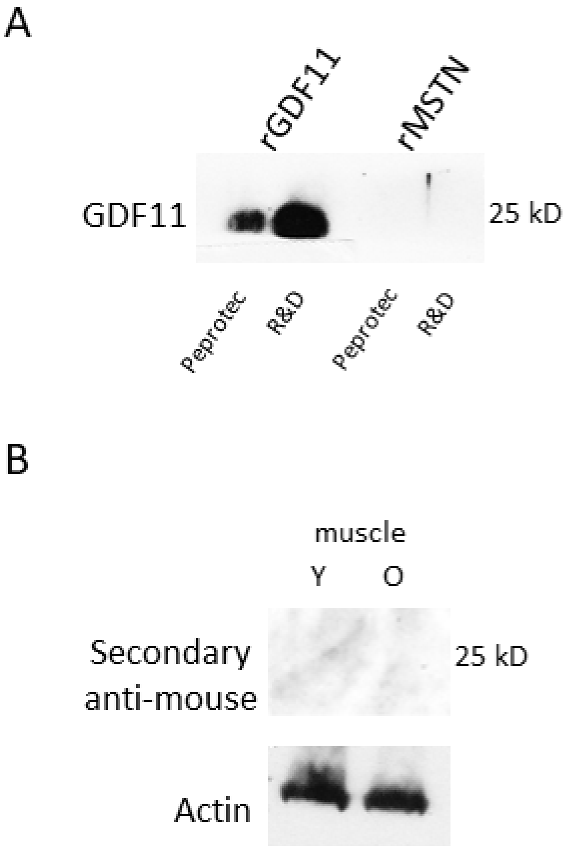

Supplementary Figure 3: Assessment of antibody specificity for GDF11.
Panel A Anti-GDF11 antibody specificity against two different commercially available (Peprotech and R&D) recombinant GDF11 (rGDF11) and rMSTN (100ng) proteins.
Panel B Analysis of the secondary anti-mouse antibody reactivity with skeletal muscle tissue extracts. Mouse monoclonal antibody does not react with native protein band at 25 kD.
